# Retrospective Analysis of Diagnostic and Prognostic Value of Serum Glypican-3 in Patients With HCV-Related Cirrhosis With Or Without HCC After Achieving SVR With DAA Treatment

**DOI:** 10.1007/s12029-025-01371-0

**Published:** 2026-01-09

**Authors:** Gian Paolo Caviglia, Marta Guariglia, Silvia Gaia, Yulia Troshina, Emanuela Rolle, Francesca Saba, Eleonora Dileo, Patrizia Carucci, Alessia Ciancio

**Affiliations:** 1 Department of Medical Sciences, University of Torino, Turin, 10126 Italy; 2 Città Della Salute E Della Scienza, Molinette Hospital, Turin, 10126 Italy

**Keywords:** Biomarker, Diagnostic accuracy, Direct acting antivirals, GPC-3, Liver cancer, Overall survival

## Abstract

**Purpose:**

Despite achieving sustained virologic response (SVR) after treatment with direct-acting antivirals (DAAs), patients with hepatitis C virus (HCV)-related cirrhosis remain at risk of hepatocellular carcinoma (HCC) development. Glypican-3 (GPC-3) is a heparan sulfate proteoglycan with oncogenic role in HCC. This study aimed to assess the diagnostic and prognostic value of serum GPC-3 in patients with HCV-related cirrhosis who achieved SVR following DAA therapy.

**Methods:**

We conducted a retrospective, observational study including 832 patients with HCV-related cirrhosis treated with DAAs between 2014 and 2024. Patients were divided into two cohorts: cohort A (*n* = 551) without HCC at enrolment and cohort B (*n* = 281) with established HCC. Serum GPC-3 was measured using a commercially available enzyme immunoassay (CanAg Glypican-3 EIA, Fujirebio Diagnostics AB, Gothenburg, Sweden).

**Results:**

We analyzed 832 single serum samples: collected at SVR12 in Cohort A and at HCC diagnosis in Cohort B. GPC-3 levels were significantly higher in patients with HCC compared to those without (95, 50–185 pg/mL vs. 48, 29–79 pg/mL; *p* < 0.001), with moderate diagnostic accuracy (AUC = 0.711). During follow-up (37, 20–51 months), GPC-3 levels did not predict the development of de novo HCC in cohort A. However, in cohort B, GPC-3 > 150 pg/mL was independently associated with reduced survival (adjusted HR = 1.68, 95% CI 1.03–2.67, *p* = 0.036).

**Conclusions:**

While GPC-3 may be of limited utility for predicting HCC occurrence in patients cured of HCV, it could represent a valuable prognostic factor able to predict survival of patients with established HCC.

**Supplementary Information:**

The online version contains supplementary material available at 10.1007/s12029-025-01371-0.

## Introduction

Hepatocellular carcinoma (HCC) is the most common primary liver malignancy and ranks as the fourth leading cause of cancer-related mortality worldwide [1]. In Western countries, HCC remains a major contributor to cancer mortality, and late-stage presentation is still common despite established surveillance programs [2, 3]. Early detection remains critical to improve survival; however, semiannual ultrasound with or without alpha-fetoprotein (AFP), has limited sensitivity for early-stage disease and does not account for individual risk stratification [3].

Cirrhosis, regardless of etiology, remains the principal risk factor for HCC development, and hepatitis C virus (HCV) infection represents one of its most frequent causes globally [4]. The introduction of direct-acting antivirals (DAAs) has revolutionized HCV therapy, achieving sustained virologic response (SVR) rates exceeding 98% and leading to substantial reductions in hepatic inflammation, fibrosis progression, and liver-related complications [5, 6]. However, HCC risk persists even after viral eradication, particularly in patients with advanced fibrosis or cirrhosis; epidemiological studies have estimated an annual HCC incidence of approximately 1.5–2.5% among patients with HCV-related cirrhosis after SVR, warranting continued surveillance in this population [7]. The risk may persist for up to a decade after SVR, underscoring the need for long-term risk assessment [8]. Importantly, this residual risk is not uniform and increases with baseline disease severity (i.e., portal hypertension and impaired liver function) [2, 7, 8]. Furthermore, additional cofactors may modulate residual HCC risk after HCV cure [9, 10], with higher annual rates reported in selected high-risk subgroups [7].

Glypican-3 (GPC-3), a membrane-bound heparan sulfate proteoglycan, is selectively overexpressed in malignant hepatocytes and enhances oncogenic signalling pathways, including Wnt/β-catenin [11]. In risk-based surveillance, baseline risk is primarily determined by liver disease severity, whereas circulating tumor biomarkers can support detection of small/subclinical HCC and reduce missed cancers by complementing imaging and informing recall strategies [12, 13]. Although GPC-3 is well established in tissue-based diagnostics, the clinical value of its circulating form remains unclear [14–17]. Previous evidence suggests that elevated circulating GPC-3 levels may correlate with tumor presence and aggressiveness [18, 19], but its diagnostic and prognostic performance in patients with HCV-related cirrhosis with or without HCC following DAA treatment has not been defined. Accordingly, we aimed to: (I) assess the diagnostic accuracy of serum GPC-3 for distinguishing HCC from cirrhosis without tumor; (II) evaluate whether baseline post-SVR GPC-3 predicts de novo HCC during the follow-up (FU); and (III) determine the prognostic value of GPC-3 in patients with established HCC.

## Methods

### Study Design and Patients

This observational, retrospective, single-center study enrolled adult patients (age ≥ 18 years) with HCV-related cirrhosis with or without HCC who achieved SVR after DAA treatment at the Liver Unit of the Città della Salute e della Scienza – Molinette Hospital of Turin between June 2014 and June 2024.

Patients included in the study belonged to two different cohorts.

- Cohort A: consecutive patients with HCV-related cirrhosis, without any prior or current history of HCC, who achieved SVR after DAA treatment and had a stored frozen serum sample available, collected 12 weeks after achieving sustained virologic response (SVR12). All the patients underwent post-SVR surveillance with semiannual clinical/laboratory FU and abdominal ultrasound every 3–6 months until HCC detection, death, or last FU.
- Cohort B: consecutive patients with HCV-related cirrhosis and established HCC, all of whom had achieved SVR before tumor diagnosis, with a frozen serum sample collected at the time of HCC diagnosis before initiation of HCC-specific therapy.

Each participant contributed one stored serum specimen for GPC-3 quantification; pre-treatment (pre-DAA) sera were not available. For all patients, we collected the principal demographic (age and sex), clinical (Child-Turcotte-Pugh [CTP] class and presence of esophageal varices), and biochemical features (alanine aminotransferase [ALT], aspartate aminotransferase [AST], γ-glutamyl transferase [γGT], platelet count, albumin, total bilirubin, international normalized ratio [INR], and AFP).

Liver cirrhosis was diagnosed based on histological assessment or by non-invasive criteria, including vibration-controlled transient elastography (FibroScan, Echosens, Paris, France) with a liver stiffness measurement > 11.9 kPa [20], and/or by indirect signs of portal hypertension, such as presence of abdominal collateral veins, platelet count < 150 × 109/L, or esophageal varices [21]. SVR to DAA therapy was defined as undetectable HCV-RNA 12 weeks after the end of treatment [22].

The diagnosis of HCC was established according to international guidelines [2], based on either histological confirmation or non-invasive imaging criteria. Specifically, HCC was diagnosed through percutaneous liver biopsy or radiologically by the presence of a liver nodule ≥ 1 cm showing typical hallmarks on dynamic contrast-enhanced imaging (arterial phase hyperenhancement followed by washout in the portal venous and/or delayed phases) on either computed tomography or magnetic resonance imaging. The Barcelona Clinic Liver Cancer (BCLC) staging system was adopted for classifying patients and allocating treatment; in some cases, a multidisciplinary approach was required to determine the most appropriate therapeutic strategy.

The study was conducted in accordance with the Declaration of Helsinki and approved by the Ethics Committee of A.O.U. Città della Salute e della Scienza di Torino—A.O. Ordine Mauriziano—A.S.L. Città di Torino (protocol code 0008732, approval date 26 January 2022). Written informed consent was obtained from all patients before enrolment.

### Study Endpoints

The primary endpoints of the study were: (1) incidence of HCC during long-term FU in patients with HCV-related cirrhosis who achieved SVR after DAA therapy (cohort A), aimed at evaluating the predictive ability of baseline serum GPC-3 levels for tumor development; and (2) OS in patients with HCV-related cirrhosis and established HCC at the time of serum sampling (cohort B), aimed at assessing the prognostic value of baseline serum GPC-3 concentrations. As secondary endpoints, we evaluated the association between baseline GPC-3 and recurrence-free survival (RFS) in patients with HCC undergoing curative-intent therapies, and progression-free survival (PFS) in patients managed with disease-control therapies.

### Serum GPC-3 Measurement

Circulating GPC-3 levels were measured in serum samples that had been stored at − 80 °C until analysis. Quantification was performed using a two-step enzyme immunoassay (CanAg Glypican-3 EIA, Fujirebio Diagnostics AB, Gothenburg, Sweden), in accordance with the manufacturer’s instructions. Absorbance was read at 450 nm using a spectrophotometric plate reader, and results were expressed in pg/mL, with a dynamic measurement range of 0–2710 pg/mL [18, 19].

### Statistical Analysis

Continuous variables were expressed as median and interquartile range (IQR), while categorical variables were reported as absolute frequency (n) and percentage (%). Normality of distribution was assessed using the D’Agostino–Pearson test. Comparisons between two independent groups were performed using the Mann–Whitney U test for continuous variables and the chi-squared (χ^2^) test for categorical variables. Correlation analysis was performed by Spearman's rank test.

The diagnostic accuracy of serum GPC-3 in distinguishing patients with and without HCC was evaluated by receiver operating characteristic (ROC) curve analysis. The area under the curve (AUC) with the corresponding 95% confidence interval (CI) was used to quantify GPC-3 performance. Logistic regression analysis was used to evaluate the association between circulating biomarkers and HCC.

In cohort A, the cumulative incidence of de novo HCC according to baseline GPC-3 levels was estimated using the Kaplan–Meier method, and differences between groups were assessed with the log-rank test. In cohort B, OS was similarly analyzed using Kaplan–Meier curves; univariate and multivariate Cox proportional hazards regression analysis was performed to identify variables associated with the risk of death. Results were reported as hazard ratios (HRs) and 95% CIs.

All statistical analyses were conducted using MedCalc (v.20.104, MedCalc Software Ltd., Ostend, Belgium). A two-sided *p*-value < 0.05 was considered statistically significant.

## Results

### Patients’ Characteristics

**Fig. 1:**
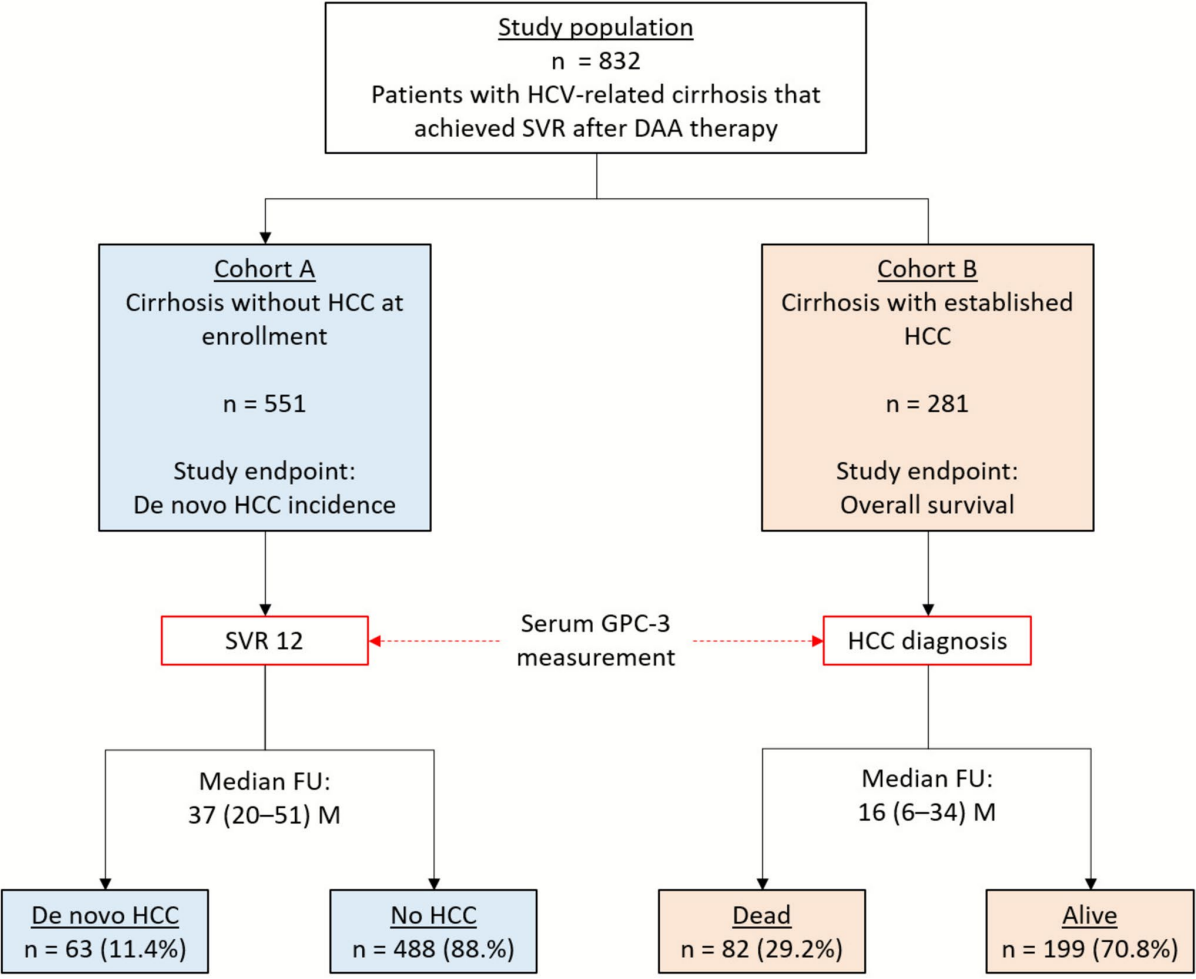
Flow chart of the study population and design. All patients had achieved sustained virologic response (SVR) after direct-acting antiviral (DAA) therapy. Serum samples were collected at SVR12 in Cohort A (without HCC) and at the time of HCC diagnosis in Cohort B (with established HCC). Primary endpoints were de novo HCC incidence and overall survival, respectively. Abbreviations: DAA, direct-acting antivirals; FU, follow-up; GPC-3, Glypican-3; HCC, hepatocellular carcinoma; M, months; SVR, sustained virologic response

Overall, 832 patients with HCV-related cirrhosis who underwent DAA treatment were included in the study; 551 patients belonged to cohort A (i.e., patients without HCC at enrolment) and 281 patients belonged to cohort B (i.e., patients with HCC at enrolment) (Fig. 1). Their main features are reported in Table 1.

**Table 1:** Characteristics of the study cohort

Variables *	Overall	Cohort A	Cohort B**	*p*-value
Patients, n (%)	832 (100%)	551 (66.2%)	281 (33.8%)	
Age (years), median (IQR)	63 (57–75)	65 (57–76)	62 (56–72)	0.007
Male sex, n (%)	544 (65.4%)	333 (61.2%)	211 (75.1%)	< 0.001
Child-Turcotte-Pugh score A, n (%)	742 (89.2%)	502 (91.1%)	240 (86.0%)	0.012
Esophageal varices, n (%)	270 (32.5%)	152 (27.6%)	118 (42.0%)	< 0.001
ALT (U/L), median (IQR)^†^	21 (17–30)	20 (16–26)	27 (18–39)	< 0.001
AST (U/L), median (IQR)	25 (21–33)	24 (21–29)	32 (24–48)	< 0.001
γGT (U/L), median (IQR)	30 (21–54)	28 (19–40)	49 (29–92)	< 0.001
Platelet count (× 10^9^/L), median (IQR)	130 (85–168)	130 (91–163)	124 (73–177)	0.537
Albumin (g/dL), median (IQR)	4.3 (4.0–4.5)	4.3 (4.1–4.6)	4.2 (3.7–4.5)	< 0.001
Total bilirubin (mg/dL), median (IQR)	0.8 (0.6–1.1)	0.7 (0.5–1.0)	0.9 (0.7–1.4)	< 0.001
INR, median (IQR)	1.13 (1.06–1.24)	1.11 (1.05–1.22)	1.14 (1.07–1.29)	0.001
AFP (ng/mL), median (IQR)	4.5 (2.8–9.7)	3.7 (2.5–5.8)	8.2 (3.5–48.2)	< 0.001
Unifocal HCC, n (%)			175 (62.3%)	
Major nodule size (mm), median (IQR)			27 (18–40)	
BCLC, 0/A/B/C			48/146/45/42	

Patients in cohort B were younger than those in cohort A (median age: 62 vs. 65 years; *p* = 0.007), exhibited a higher proportion of males (75.1% vs. 61.2%; *p* < 0.001), and had more advanced liver dysfunction. Specifically, cohort B showed a higher prevalence of esophageal varices (42.0% vs. 27.6%; *p* < 0.001), reduced rates of CTP class A (86.0% vs. 91.1%;* p* = 0.012), and significantly lower serum albumin levels (4.2 vs. 4.3 g/dL; *p* < 0.001). Most of the patients from cohort B had a diagnosis of early tumor (BCLC 0/A = 194; 69.0%); overall, 175 out of 281 (62.3%) had a unifocal HCC.

### Diagnostic Accuracy of GPC-3 for HCC Detection

**Fig. 2:**
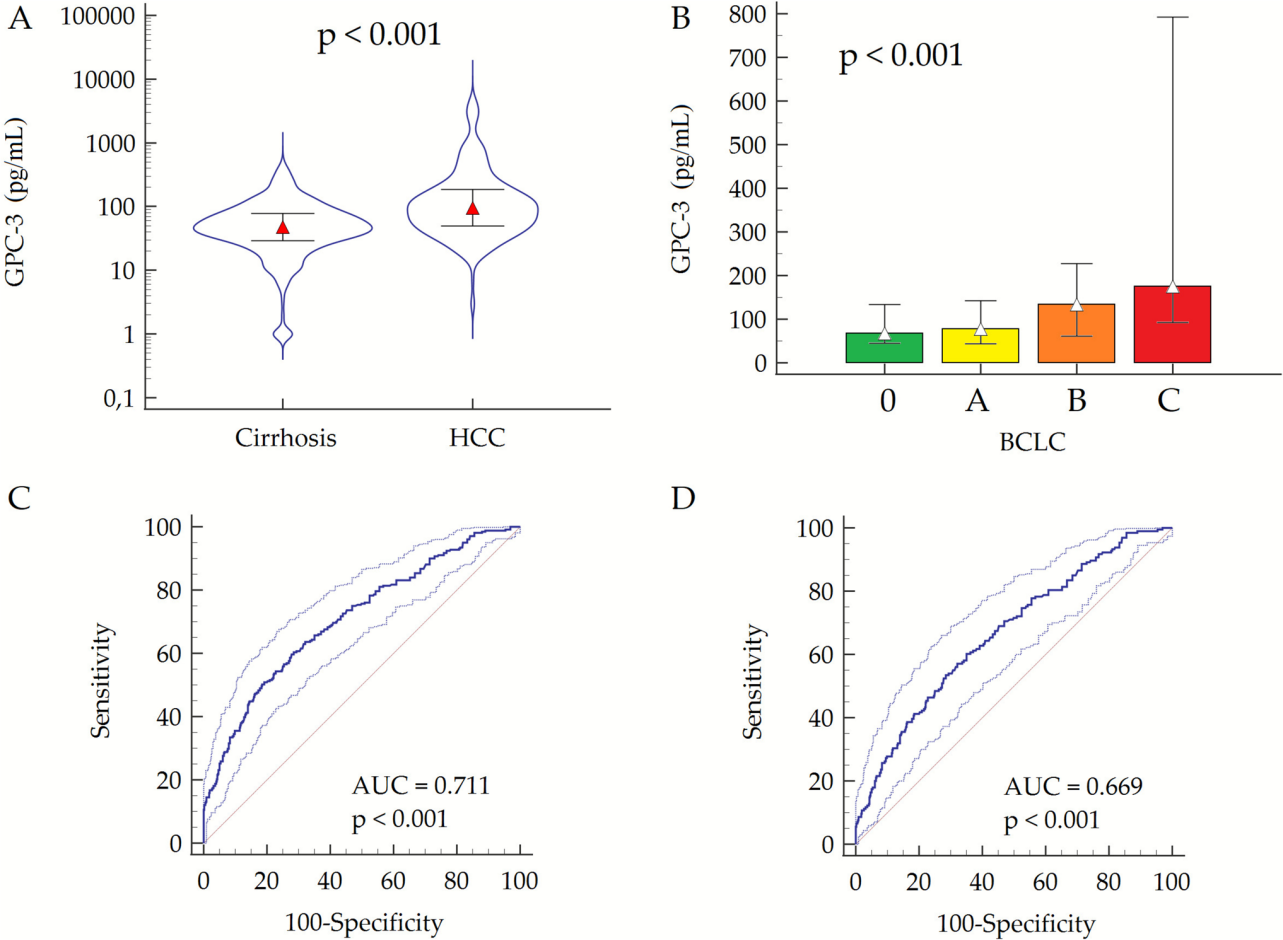
Median circulating GPC-3 levels in patients with cirrhosis compared to those with HCC (**A**), according to BCLC stage (**B**), and diagnostic performance for HCC detection (**C**) and early HCC detection (**D**). Median GPC-3 values in Fig. 1A are depicted on a Log scale; *p*-value has been calculated by Mann–Whitney test (**A**) or by Kruskal–Wallis test (**B**). Abbreviations: AUC, area under the curve; BCLC, Barcelona Clinic Liver Cancer; GPC-3, glypican-3; HCC, hepatocellular carcinoma

We first report GPC-3 distributions in cirrhosis without HCC (cohort A) and with HCC (cohort B), followed by diagnostic performance (ROC) in the overall population. Median serum GPC-3 values were significantly higher in patients from cohort B than those from cohort A (95, 50–185 pg/mL vs. 48, 29–79 pg/mL; *p* < 0.001) (Fig. 2A). In the overall population (*n* = 832), we observed a moderate direct correlation with AFP (*r*_*s*_ = 0.357, 95% CI 0.291–0.420; *p* < 0.001), a weak direct correlation between GPC-3 levels and ALT (*r*_*s*_ = 0.112, 95% CI 0.045–0.179; *p* = 0.001), AST (*r*_*s*_ = 0.168, 95% CI 0.100–0.233; *p* < 0.001), and γGT (*r*_*s*_ = 0.183, 95% CI 0.116–0.248; *p* < 0.001), as well as a weak inverse correlation with albumin (*r*_*s*_ = −0.075, 95% CI −0.142–—0.007; *p* = 0.032). No significant correlation was observed between GPC-3 and age (*r*_*s*_ = 0.059, 95% CI −0.009–0.126; *p* = 0.091), platelet count (*r*_*s*_ = −0.009, 95% CI −0.078–0.059; *p* = 0.780), INR (*r*_*s*_ = 0.052, 95% CI −0.017–0.121; *p* = 0.144) or total bilirubin (*r*_*s*_ = 0.062, 95% CI −0.006–0.130; *p* = 0.072) (Supplementary Fig. 1). Furthermore, we did not observe any differences in serum GPC-3 values according to sex (*p* = 0.240).

Among patients from cohort B, GPC-3 levels exhibited a weak correlation with the size of the major HCC nodule (*r*_*s*_ = 0.201, 95% CI 0.085–0.312; *p* = 0.001), and were significantly higher in patients with multifocal HCC than those with unifocal tumor (123, 69–226 pg/mL vs. 74, 43–149 pg/mL; *p* < 0.001). Additionally, GPC-3 concentration distinctly increased with advancing BCLC stage: 68, 46–134 pg/mL in BCLC 0, 77, 44–142 pg/mL in BCLC A, 134, 61–227 pg/mL in BCLC B, and 175, 93–792 in BCLC C (*p* < 0.001) (Fig. 2B). Similarly, AFP values stepwise increased according to BCLC stage (Supplementary Table 1 and Supplementary Fig. 2).

By ROC curve analysis, we observed a moderate diagnostic accuracy for the discrimination between patients with cirrhosis (cohort A; *n* = 551) and those with HCC (cohort B; *n* = 281) (AUC = 0.711, 95% CI 0.679–0.742) (Fig. 2C). Diagnostic performance remained comparable when the analysis was restricted to patients with early-stage HCC (BCLC stage 0/A; *n* = 194), yielding an AUC of 0.669, 95% CI 0.634–0.703 (DeLong test: *p* = 0.155) (Fig. 2D). For comparison, AFP achieved an AUC of 0.707 (95% CI, 0.672–0.740) for overall HCC detection and 0.641 (95% CI, 0.602–0.679) for early-stage HCC. Notably, in multivariable logistic regression, GPC-3 was independently associated with HCC (OR = 1.01, 95% CI 1.00–1.01; *p* < 0.001), whereas AFP was not (OR = 1.00, 95% CI 0.99–1.00; *p* = 0.115).

### Stratification of the Risk of HCC Development According to GPC-3

We next evaluated the predictive value of baseline (SVR12) GPC-3 for de novo HCC in cohort A. During a median FU of 37 (20–51) months, 63 (11.4%) patients from cohort A developed HCC (incidence rate: 3.83 cases per 100 person/years). Patients’ characteristics at SVR12, stratified by HCC development during FU, are summarized in Supplementary Table 2.

At the time of HCC diagnosis, the majority of patients presented with early-stage tumors (BCLC 0/A, *n* = 38; 60.3%), while 13 (20.6%) patients were diagnosed with intermediate-stage disease (BCLC B), and 12 (19.0%) with advanced-stage HCC (BCLC C). Overall, 35 out of 63 patients (55.6%) had a unifocal tumor, and the median diameter of the largest nodule was 32 (18–45) mm. Incident HCC occurred under the semiannual ultrasound-based surveillance; nonetheless, given the retrospective design, a minority of patients may have missed scheduled ultrasounds, which could have contributed to the observed stage distribution.

**Fig. 3:**
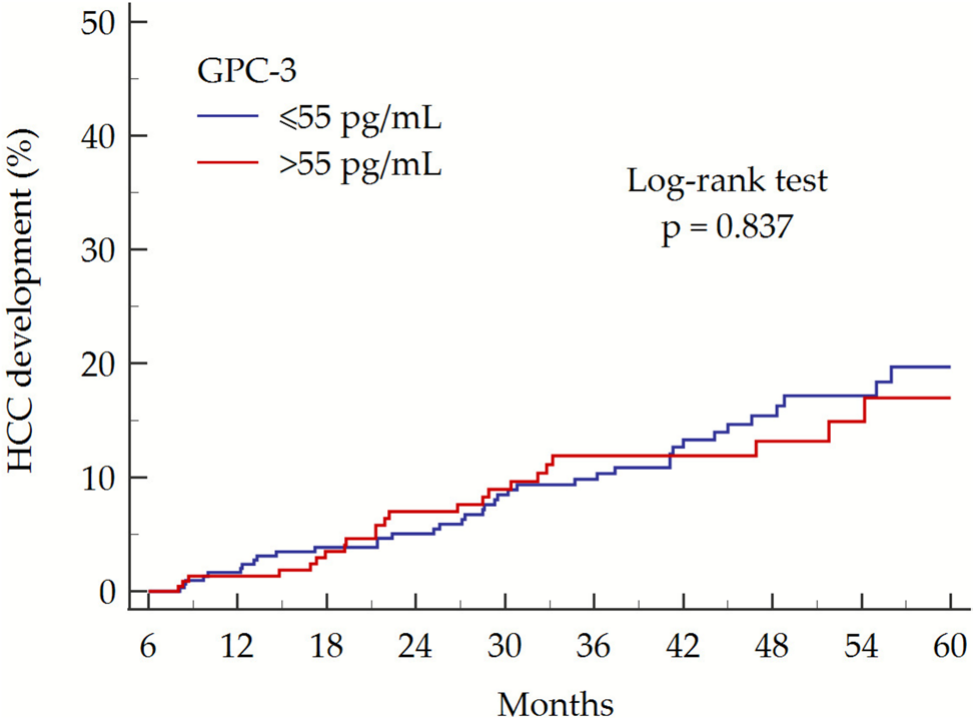
HCC incidence in patients with cirrhosis (cohort A) according to baseline (SVR12) serum GPC-3 > 55 pg/mL. Abbreviations: GPC-3, glypican-3; HCC, hepatocellular carcinoma

To investigate the predictive ability of baseline serum GPC-3 levels for HCC development, patients were stratified according to serum GPC-3 levels > 55 pg/mL, corresponding to the upper reference limit derived from measurements in 243 healthy blood donors [23]. Kaplan–Meier survival analysis revealed no significant difference in HCC incidence between patients with GPC-3 levels ≤ 55 pg/mL (39 incident cases [12.2%] among 320 patients) and those with levels > 55 pg/mL (24 incident cases [10.4%] among 231 patients) (log-rank test: *p* = 0.837; Fig. 3).

### Prediction of Overall Survival According to GPC-3

Finally, we assessed the prognostic value of diagnosis-time GPC-3 for overall survival in cohort B. Patients with HCC at baseline (cohort B) had a median FU of 16 (6–34) months. A total of 82 (29.2%) patients died during the FU; median OS was 62 (45–99) months. The 1-, 3-, and 5-year OS rates were 90.8% (109/120), 63.7% (65/102), and 43.4% (36/83), respectively. As expected, overall survival decreased progressively with advancing BCLC stage at diagnosis (Supplementary Table 3; Supplementary Fig. 3).

**Fig. 4:**
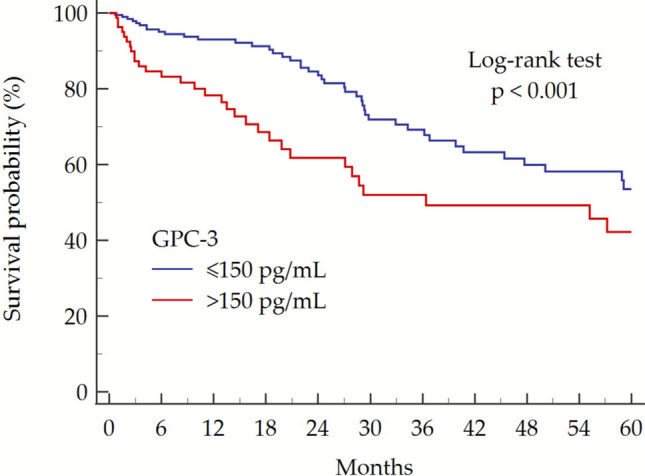
OS in patients with HCC (cohort B) according to baseline serum GPC-3 > 150 pg/mL. Abbreviations: GPC-3, glypican-3

To evaluate the prognostic significance of baseline serum GPC-3 concentrations, patients were stratified according to a serum GPC-3 threshold of > 150 pg/mL, a cut-off that was previously identified in a cohort of patients with HCC and cirrhosis of various etiologies [24]. Kaplan–Meier survival analysis showed that patients with baseline GPC-3 > 150 pg/mL had significant reduced OS compared to those with levels ≤ 150 pg/mL (36, 95% CI 21–78 months vs. 68, 95% CI 48–103 months, respectively; Log-rank test: *p* = 0.012) (Fig. 4). Consistently, 1-, 3-, and 5-year overall survival rates were 96.3% (79/82), 68.7% (46/67), and 46.2% (24/52), respectively, in patients with GPC-3 ≤ 150 pg/mL, compared to 78.9% (30/38), 54.3% (19/35), and 38.7% (12/31) in those with GPC-3 > 150 pg/mL.

Cox proportional-hazards regression revealed that GPC-3 > 150 pg/mL was significantly associated with reduced OS (HR = 1.76, 95% CI 1.12–2.74; *p* = 0.013). In multivariate analysis adjusted for age, sex, tumor stage (BCLC) and HCC treatment, GPC-3 > 150 pg/mL remained an independent predictor of poorer survival (aHR = 1.68, 95% CI 1.03–2.67, *p* = 0.036) (Table 2). Results were consistent in a sensitivity analysis replacing BCLC with ALBI and explicit tumor-burden covariates (nodule size, multifocality, extrahepatic spread) (Supplementary Table 4).

**Table 2:** Univariate and multivariate Cox proportional-hazard regression analysis of OS predictors

Covariates	Univariate HR, 95% CI	*p*-value	Multivariate aHR, 95% CI	*p*-value
GPC-3 > 150 pg/mL	1.76, 1.12–2.74	0.013	1.68, 1.03–2.67	0.036
Age (years)	1.00, 0.97–1.02	0.748	1.01, 0.98–1.03	0.605
Male sex	1.37, 0.83–2.26	0.214	1.13, 0.65–1.96	0.675
BCLC stage	2.18, 1.73–2.76	< 0.001	2.08, 1.57–2.75	< 0.001
Curative-intent therapy*	0.18, 0.10–0.31	< 0.001	0.19, 0.11–0.34	< 0.001
Disease-control therapy**	0.57, 0.33–0.98	0.043	0.28, 0.15–0.53	< 0.001

### Prognostic Impact of GPC-3 by Treatment Intent

To further explore the prognostic significance of GPC-3, survival analyses were stratified by treatment intent and conducted separately in patients receiving curative-intent therapies (resection/ablation) and those managed with disease-control therapies (endovascular or systemic treatment). In patients who underwent curative-intent therapy, median OS was 102.5 (95% CI, 78.2–113.9) months and median RFS was 27.5 (95% CI, 23.1–43.1) months; baseline GPC-3 was not associated with RFS (aHR, 0.87, 95% CI, 0.50–1.50; *p* = 0.608) (Supplementary Table 5). Conversely, in patients treated with disease-control therapies, median OS was 29.8 (95% CI 27.1–58.9) months and median PFS was 14.4 (95% CI 8.3–25.2) months; GPC-3 > 150 pg/mL was significantly and independently associated with shorter PFS (aHR, 2.61, 95% CI 1.27–5.38; *p* = 0.009), irrespective of age, sex, BCLC stage, and treatment class (endovascular vs systemic) (Supplementary Table 6).

## Discussion

In this large cohort of patients with HCV-related cirrhosis who achieved SVR following DAA therapy, we observed that circulating GPC-3 levels were significantly elevated in patients with HCC and correlated with tumor burden and disease stage. Furthermore, serum GPC-3 concentrations > 150 pg/mL were independently associated with reduced OS in patients with HCC; its prognostic value appeared even more pronounced in patients with advanced tumors who were candidates for disease-control therapies. Conversely, baseline GPC-3 levels at SVR12 did not predict de novo HCC development in patients without baseline malignancy, thereby limiting its application as a predictive single-time-point biomarker in the surveillance setting.

In the post-DAA era, achieving SVR confers substantial clinical benefit, with marked improvements in liver outcomes and overall survival. Nevertheless, a residual risk of HCC persists among patients with cirrhosis or advanced fibrosis (F3–F4), supporting the need for ongoing long-term surveillance after viral cure. By contrast, patients without advanced fibrosis can generally be discharged from specialized FU, whereas surveillance in those with F3 fibrosis should be individualized according to fibrosis stage and coexisting risk factors [2, 25].

The diagnostic performance of GPC-3 observed in our cohort (AUC = 0.711) broadly aligns with data from an earlier meta-analysis, which reported an AUC of 0.82, based on 12 studies evaluating GPC-3 in serum for HCC diagnosis [26]. However, the same study pointed out significant methodological heterogeneity, including biases related to patient selection and assay variability, which limit the generalizability of the pooled estimates. Our data support the notion that GPC-3 has moderate standalone diagnostic performance, though it may achieve greater utility when integrated into multimarker panels [16, 20]. In our cohort, GPC-3 and AFP provided comparable diagnostic accuracy for HCC (AUC 0.711 and 0.707, respectively), with GPC-3 remaining independently associated with HCC in multivariable models, while AFP did not. Notably, the two markers showed a moderate positive correlation, suggesting shared biological information, but also indicating that GPC-3 offers partially independent diagnostic information beyond AFP. This partial independence is consistent with the moderate correlation observed between GPC-3 and AFP, supporting the notion that the two biomarkers capture distinct, though partly overlapping, biological processes.

Although the present study was not designed to evaluate multi-marker scores or test combinations, we acknowledge that composite approaches may enhance diagnostic and prognostic performance beyond single biomarkers. Our findings, showing that GPC-3 is independently associated with both HCC detection and OS, support the hypothesis that GPC-3 might provide additive value when incorporated into multi-marker algorithms. Future prospective studies with harmonized assessment of biomarkers, including but not limited to AFP and PIVKA-II are warranted to determine whether GPC-3 may contribute to the development of new composite models for HCC detection and prognostication, and to explore its potential value in risk stratification when assessed in longitudinal or alternative clinical settings, acknowledging that such association was not observed in our cohort.

A relevant biological aspect to consider is the influence of liver disease etiology on GPC-3 expression and secretion. Shimizu et al. demonstrated that plasma GPC-3 levels and tumoral expression are significantly higher in patients with HCV-related HCC compared to those with hepatitis B virus-related or non-viral HCC, indicating that viral background may modulate GPC-3 regulation [27]. While our entire cohort included only patients who had achieved HCV eradication and were therefore free of active viral replication, prior hepatic injury and regenerative activity may nonetheless have influenced baseline GPC-3 levels. Furthermore, the study by Abdul-Al et al. raises concerns regarding the specificity of GPC-3 in patients with prior HCV-related liver damage, reporting that strong GPC-3 immunoreactivity was observed in 83% of biopsies from patients with high-grade chronic hepatitis C, in the absence of malignancy [28]. This supports the hypothesis that GPC-3 may also be expressed by regenerating hepatocytes or during early dysplastic changes, thereby reducing its specificity in surveillance contexts, particularly among patients with a history of chronic HCV infection.

Nonetheless, it should be noted that GPC-3 was measured only once at SVR12 in cohort A, without longitudinal assessment. Although baseline levels did not predict subsequent HCC development, the lack of serial measurements limits our ability to fully evaluate whether GPC-3 changes over time may have predictive value. Notably, Ricco et al. showed that dynamic increases in PIVKA-II anticipated HCC onset in at-risk patients [29]; while GPC-3 may follow a different biological pattern than PIVKA-II, this hypothesis deserves further exploration before its role in risk stratification is definitively excluded. Accordingly, the predictive performance of circulating GPC-3 should be revisited in future studies using longitudinal sampling and clinically synchronous time-points. Finally, the study was not designed to include additional non-standard biomarkers such as PIVKA-II, and our analyses were therefore limited to GPC-3 and AFP, the latter representing the established benchmark in current clinical guidelines.

Residual HCC risk after SVR is heterogeneous and is influenced by baseline liver disease severity and markers of portal hypertension. In a risk-stratified surveillance framework, integrating clinical severity metrics with biomarker information may help tailor FU intensity, provided that markers are evaluated at clinically meaningful time points. Differences by presumed acquisition route likely mirror underlying risk modifiers (age/duration of infection, genotype distribution, liver disease severity, HIV coinfection) rather than acting as independent determinants of post-SVR HCC risk. This perspective is consistent with contemporary Western cohorts in which age and liver function/surrogates of portal hypertension, rather than transmission route, drive most of the residual risk after cure [30].

Against this background, our findings support the role of GPC-3 as a valuable prognostic marker in patients with established HCC. Kaplan–Meier and multivariate Cox analyses demonstrated that serum GPC-3 levels > 150 pg/mL were independently associated with reduced OS, even after adjusting for tumor stage and therapy. Notably, the prognostic effect of baseline GPC-3 appeared context-dependent, being null for post-curative recurrence (RFS) but more pronounced among patients managed with disease-control therapies, in whom higher GPC-3 independently predicted shorter PFS (aHR 2.61, *p* = 0.009). This clinical observation is biologically plausible since GPC-3 is known to actively contribute to HCC progression through modulation of several oncogenic signalling pathways [31]. GPC-3 promotes tumor growth by enhancing canonical Wnt/β-catenin signalling, leading to increased expression of oncogenes such as c-Myc and Cyclin D1 [32, 33]. Additionally, GPC-3 mediates epithelial–mesenchymal transition (EMT) and invasiveness via activation of the ERK signalling cascade, and its expression has been associated with vascular invasion and advanced BCLC stages [34]. In vitro studies have shown that HCC cells with high GPC-3 expression display greater invasive potential and EMT-like changes compared to those with lower GPC-3 levels [35]. Taken together, these data suggest that elevated serum GPC-3 may reflect not only tumor presence but also a more aggressive biological phenotype, thus offering a plausible explanation for the worse prognosis observed in patients with high GPC-3 circulating levels.

In conclusion, while GPC-3 may have limited value as a single time-point measurement for predicting HCC development in patients with HCV-related cirrhosis after SVR to DAA therapy, our findings support its potential role as a diagnostic and prognostic biomarker, particularly for risk stratification and clinical decision-making after HCC diagnosis. Future studies should explore longitudinal variations in serum GPC-3 concentrations in relation to the different therapeutic strategies currently available for the treatment of patients with HCC.

## Supplementary Information

Below is the link to the electronic supplementary material.

Supplementary file1 (DOCX 384 KB)

## Data Availability

The data presented in this study are available on request from the corresponding author.
