# Supplementary material for: Retrospective Analysis of Diagnostic and Prognostic Value of Serum Glypican-3 in Patients With HCV-Related Cirrhosis With Or Without HCC After Achieving SVR With DAA Treatment
Source: J Gastrointest Cancer. 2026 Jan 9;57(1):6. doi: 10.1007/s12029-025-01371-0 (PMC12789133; doi:10.1007/s12029-025-01371-0)

**Supplementary Table 1.** Median circulating AFP levels in patients with HCC according to BCLC stage.

|  | **BCLC** | | | |  |
| --- | --- | --- | --- | --- | --- |
|  | **0** | **A** | **B** | **C** | ***p*-value** |
| AFP (ng/mL),  median (IQR) | 4.7 (2.3–16.8) | 6.9 (3.2–16.7) | 15.6 (5.3–103.6) | 222.3 (23.6–2000) | <0.001 |

The *p*-value was calculated using the Kruskal-Wallis non-parametric test.

Abbreviations: AFP, alpha-fetoprotein; BCLC, Barcelona Clinic Liver Cancer; IQR, interquartile range.

**Supplementary Table 2.** Characteristics of patients from cohort A stratified by HCC development during the FU.

| **Variables *** | **No HCC** | **HCC during FU** | ***p*-value** |
| --- | --- | --- | --- |
| Patients, n (%) | 488 (88.6%) | 63 (11.4%) |  |
| Age (years), median (IQR) | 64 (57–76) | 67 (60–77) | 0.119 |
| Male sex, n (%) | 290 (59.4%) | 43 (68.3%) | 0.178 |
| Child-Turcotte-Pugh score A, n (%) | 454 (93.0%) | 48 (76.2%) | <0.001 |
| Esophageal varices, n (%) | 119 (24.4%) | 33 (52.4%) | <0.001 |
| ALT (U/L), median (IQR) | 20 (16–25) | 21 (18–28) | 0.037 |
| AST (U/L), median (IQR) | 24 (20–29) | 29 (24–38) | <0.001 |
| γGT (U/L), median (IQR) | 28 (18–38) | 30 (25–54) | 0.002 |
| Platelet count (×10^9^/L), median (IQR) | 130 (96–167) | 102 (73–136) | 0.001 |
| Albumin (g/dL), median (IQR) | 4.3 (4.2–4.6) | 4.2 (3.8–4.3) | <0.001 |
| Total bilirubin (mg/dL), median (IQR) | 0.7 (0.5–0.9) | 1.1 (0.6–1.7) | <0.001 |
| INR, median (IQR) | 1.11 (1.04–1.20) | 1.21 (1.11–1.34) | <0.001 |
| AFP (ng/mL), median (IQR) | 3.6 (2.4–5.5) | 4.9 (3.2–13.5) | <0.001 |

* Continuous variables were reported as median and IQR, while categorical variables as number and percentage.

Abbreviations: AFP, alpha-fetoprotein; ALT, alanine aminotransferase; AST, aspartate aminotransferase; γGT, γ-glutamyl transferase; HCC, hepatocellular carcinoma; INR, international normalized ratio; IQR, interquartile range; n, number.

**Supplementary Table 3.** Overall survival by BCLC stage at diagnosis in Cohort B: median OS (months) and hazard ratios.

| **BCLC** | **Median OS (95%CI)** | **HR (95% CI)** |
| --- | --- | --- |
| 0/A | 98.8 (59.1–113.9) | Ref. |
| B | 55.2 (27.1–61.5) | 2.12 (1.14–3.96) |
| C | 17.1 (4.3–28.4) | 5.98 (2.67–13.40) |

HRs are reported with 95% CIs; reference category: BCLC 0/A. HR were calculated by Cox regression analysis.

Abbreviations: BCLC, Barcelona Clinic Liver Cancer; CI, confidence interval; HR, hazard ratio.

**Supplementary Table 4**. Sensitivity analysis of overall survival predictors with ALBI and tumor burden covariates.

| **Covariates** | **Univariate**  **HR, 95% CI** | ***p*-value** | **Multivariate**  **aHR, 95% CI** | ***p*-value** |
| --- | --- | --- | --- | --- |
| GPC-3 >150 pg/mL | 1.76, 1.12–2.74 | 0.013 | 1.64, 1.01–2.67 | 0.048 |
| Age (years) | 1.00, 0.97–1.02 | 0.748 | 1.01, 0.99–1.04 | 0.342 |
| Male sex | 1.37, 0.83–2.26 | 0.214 | 1.45, 0.82–2.57 | 0.207 |
| ALBI score | 1.53, 1.06–2.21 | 0.024 | 1.36, 0.92–2.00 | 0.120 |
| Multifocal HCC | 2.07, 1.34–3.22 | 0.001 | 1.32, 0.80–2.18 | 0.271 |
| Major nodule > 3 cm | 2.72, 1.73–4.29 | <0.001 | 2.46, 1.46–4.16 | <0.001 |
| Extrahepatic spread | 1.57, 0.63–3.89 | 0.334 | 1.46, 0.54–3.92 | 0.452 |
| Curative-intent treatment* | 0.18, 0.10–0.31 | <0.001 | 0.17, 0.09–0.31 | <0.001 |
| Disease control treatment** | 0.57, 0.33–0.98 | 0.043 | 0.30, 0.16–0.57 | <0.001 |

The ALBI score has been included in the analyses as a continuous variable.

ALBI score was calculated according to the original formula (Log Total Bilirubin (µmol/L) * 0.66) + (Albumin (g/L) * −0.085) reported in Johnson PJ et al J Clin Oncol 2015.

* Liver resection, ablation, transplant.

** Arterial locoregional therapy, systemic therapy.

Abbreviations: aHR, adjusted hazard ratio; ALBI, albumin-bilirubin; CI, confidence interval; GPC-3, glypican-3; HCC, hepatocellular carcinoma; HR, hazard ratio.

**Supplementary Table 5.** Multivariate Cox proportional-hazard regression analysis of RFS predictors in patients undergoing curative-intent therapies as first line treatment.

| **Covariates** | **Multivariate**  **aHR, 95% CI** | ***p*-value** |
| --- | --- | --- |
| GPC-3 >150 pg/mL | 0.87, 0.50–1.50 | 0.608 |
| Age (years) | 1.00, 0.98–1.03 | 0.791 |
| Male sex | 0.94, 0.55–1.60 | 0.821 |
| BCLC stage | 1.48, 1.03–2.13 | 0.036 |
| Curative-intent treatment* | 2.15, 0.86–5.39 | 0.104 |

The BCLC stage has been included in the analyses as a continuous variable.

* ablation vs liver resection.

Abbreviations: aHR, adjusted hazard ratio; BCLC, Barcelona Clinic Liver Cancer; CI, confidence interval; GPC-3, glypican-3; HCC, hepatocellular carcinoma; HR, hazard ratio; RFS, recurrence-free survival.

**Supplementary Table 6.** Multivariate Cox proportional-hazard regression analysis of PFS predictors in patients undergoing disease-control therapies as first line treatment.

| **Covariates** | **Multivariate**  **aHR, 95% CI** | ***p*-value** |
| --- | --- | --- |
| GPC-3 >150 pg/mL | 2.61, 1.27–5.38 | 0.009 |
| Age (years) | 1.00, 0.97–1.04 | 0.879 |
| Male sex | 1.64, 0.68–3.92 | 0.269 |
| BCLC stage | 1.18, 0.85–1.65 | 0.789 |
| disease-control treatment* | 1.10, 0.54–2.24 | 0.789 |

The BCLC stage has been included in the analyses as a continuous variable.

* systemic therapy vs endovascular treatment.

Abbreviations: aHR, adjusted hazard ratio; BCLC, Barcelona Clinic Liver Cancer; CI, confidence interval; GPC-3, glypican-3; HCC, hepatocellular carcinoma; HR, hazard ratio; PFS, progression-free survival.

**Supplementary Figure 1.** Heat-map of pairwise correlations between clinical chemistry parameters and serum biomarkers in HCC patients.

**
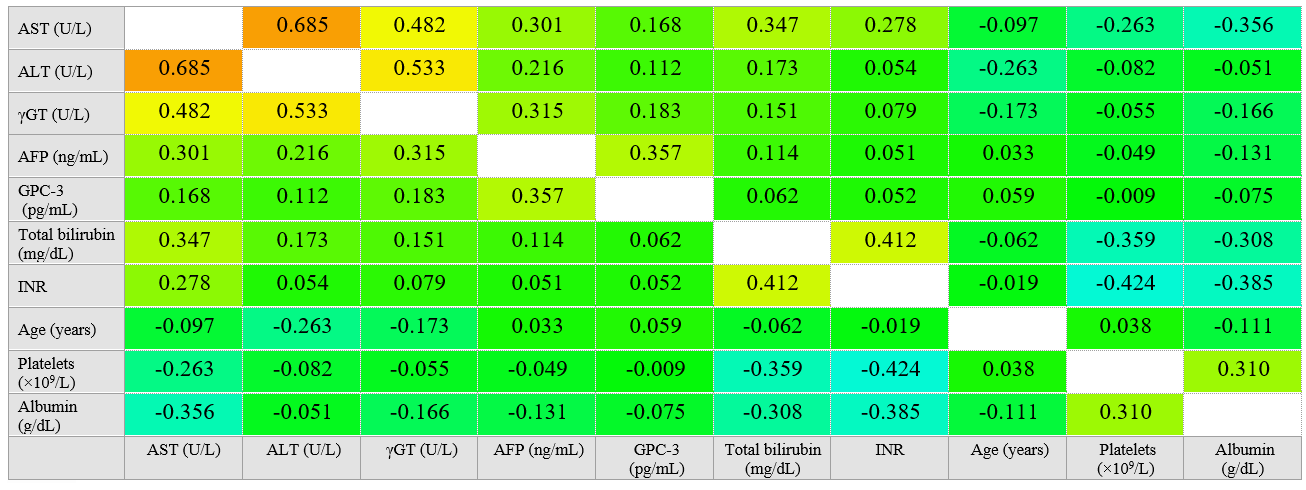
**

Correlation coefficients were calculated using Spearman correlation analysis.

Abbreviations: AFP, alpha-fetoprotein; ALT, alanine aminotransferase; AST, aspartate aminotransferase; γGT, γ-glutamyl transferase; GPC-3, glypican-3; HCC, hepatocellular carcinoma; INR, international normalized ratio.

**Supplementary Figure 2.** Median circulating AFP levels in patients with HCC according to BCLC stage.

**
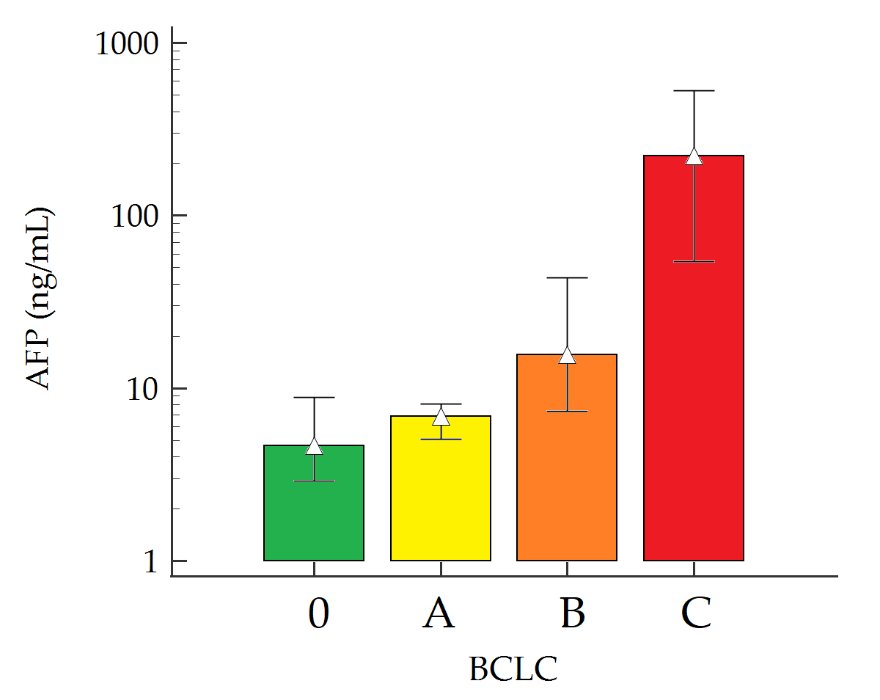
**

**Supplementary Figure 3.** Kaplan–Meier overall survival curves by BCLC stage at diagnosis in Cohort B. Abbreviations: BCLC, Barcelona Clinic Liver Cancer.


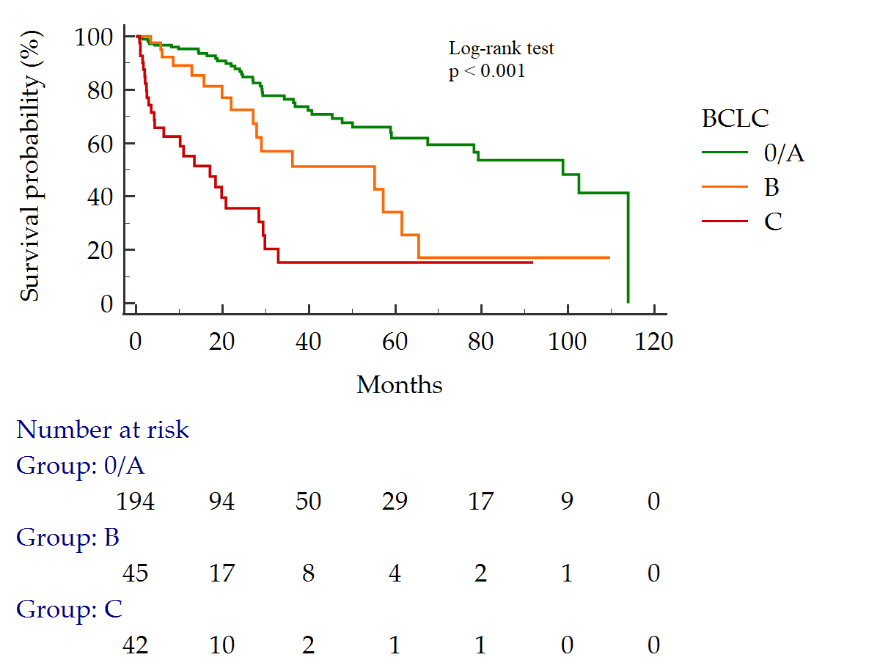

Supplement: Supplementary file 1 — Supplementary file1 (DOCX 384 KB) [file 12029_2025_1371_MOESM1_ESM.docx]
